# Supplementary material for: The AMPK agonist 5‐aminoimidazole‐4‐carboxamide ribonucleotide (AICAR), but not metformin, prevents inflammation‐associated cachectic muscle wasting
Source: EMBO Mol Med. 2018 May 29;10(7):e8307. doi: 10.15252/emmm.201708307 (PMC6034131; doi:10.15252/emmm.201708307)
Supplement: Supplementary file 1 — Appendix [file EMMM-10-e8307-s001.pdf]

Appendix

The AMPK agonist 5-Aminoimidazole-4-carboxamide ribonucleotide (AICAR), but not metformin, prevents inflammation-associated cachectic muscle wasting

Derek T. Hall[1,2], Takla Griss[2,4], Jennifer F. Ma[1,2], Brenda Janice Sanchez[1,2], Jason Sadek[1,2], Anne Marie K. Tremblay[1,2], Souad Mubaid[1,2], Amr Omer[1,2], Rebecca J. Ford[5], Nathalie Bedard[6], Arnim Pause[1,2], Simon S. Wing[6], Sergio Di Marco[1,2], Gregory R. Steinberg[5], Russell G. Jones[2,4], and Imed-Eddine Gallouzi[1,2,3*]

Table of Content … 1

Appendix Figure S1. Overexpression of AMPKα increases C2C12 sensitivity to AICAR … 2

Appendix Figure S2. Consecutive daily AICAR or metformin treatment does not affect body weight or muscle mass. … 3

Appendix Figure S3. Single doses of AICAR or metformin alone do not affect body weight or muscle mass. … 4

Appendix Table S1. qPCR Primer Sequences … 5

Appendix Table S2. Figure 1 Significance p-values … 6

Appendix Table S3. Figure 2 Significance p-values … 7

Appendix Table S4. Figure 3 Significance p-values … 8

Appendix Table S5. Figure 4 Significance p-values … 8

Appendix Table S6. Figure 5 Significance p-values … 9-10

Appendix Table S7. Figure 6 Significance p-values … 11

Appendix Table S8. Figure 7 Significance p-values … 12-14

Appendix Table S9. Figure 8 Significance p-values … 15

Appendix Table S10. Figure 9 Significance p-values … 15

Appendix Table S11. Figure EV3 Significance p-values … 16

Appendix Table S12. Figure EV4 Significance p-values … 16

Appendix Table S13. Figure EV5 Significance p-values … 17

Appendix Table S14. Table 1 Significance p-values … 18

Appendix Table S14. Table 2 Significance p-values … 19

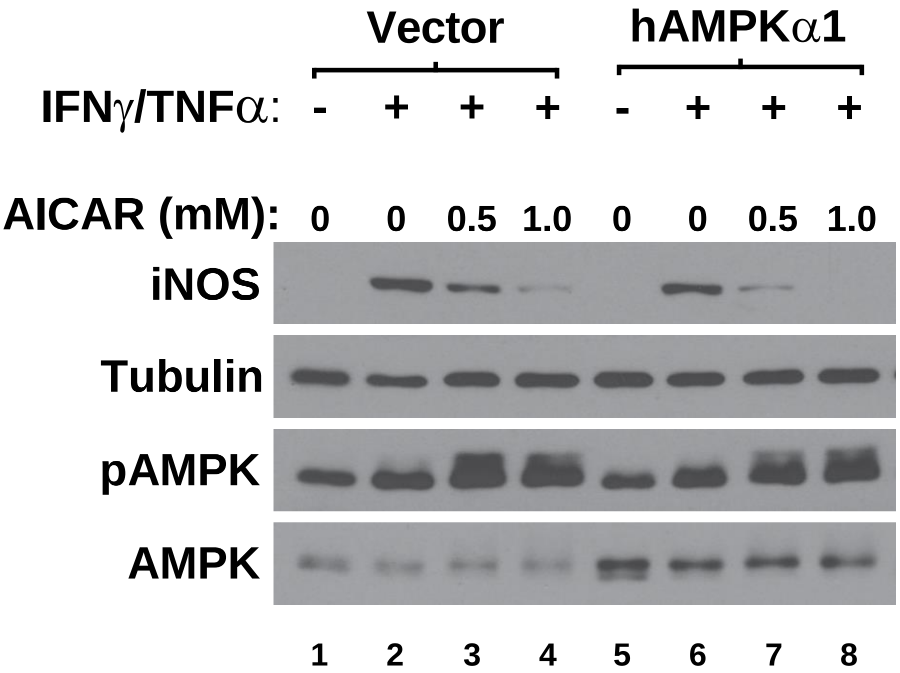

Appendix Figure S1. Overexpression of AMPKα increases C2C12 sensitivity to AICAR.

C2C12 myoblast cells were transfected with constructs expressing human AMPKα1 (hAMPKα1) or an empty vector negative control (Vector). Cells were then treated with IFNγ/TNFα and the indicated doses of AICAR. Western blot analysis performed for protein levels of iNOS, tubulin, and phosphor-Thr172-AMPK (pAMPK), and total AMPK.

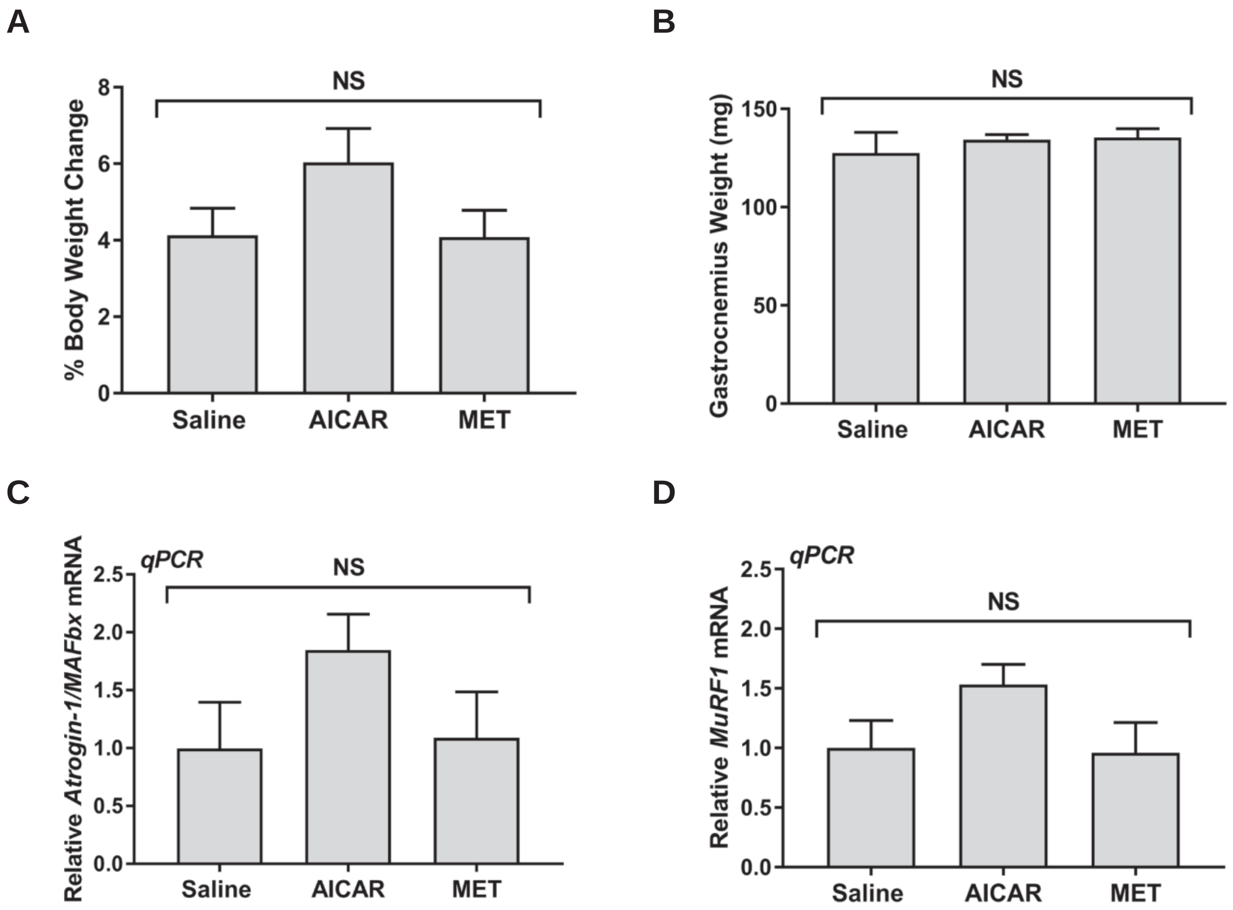

Appendix Figure S2. Consecutive daily AICAR or metformin treatment does not affect body weight or muscle mass.

BALB/C mice were intraperitoneally injected daily with AICAR (500mg kg^{-1} day^{-1}), metformin (250mg kg^{-1} day^{-1}), or an equivalent volume of saline for 7-9 days.

(A) Percent body weight change.

(B) Gastrocnemius muscle weights.

(C-D) RT-qPCR analysis of Atrogin-1/MAFbx (C) and MuRF1 (D) mRNA expression from the tibialis anterior.

Data information: Results are derived from four mice per cohort (n = 4) and error bars represent the SEM. Significance between means was determined using ANOVA. NS, not significant.

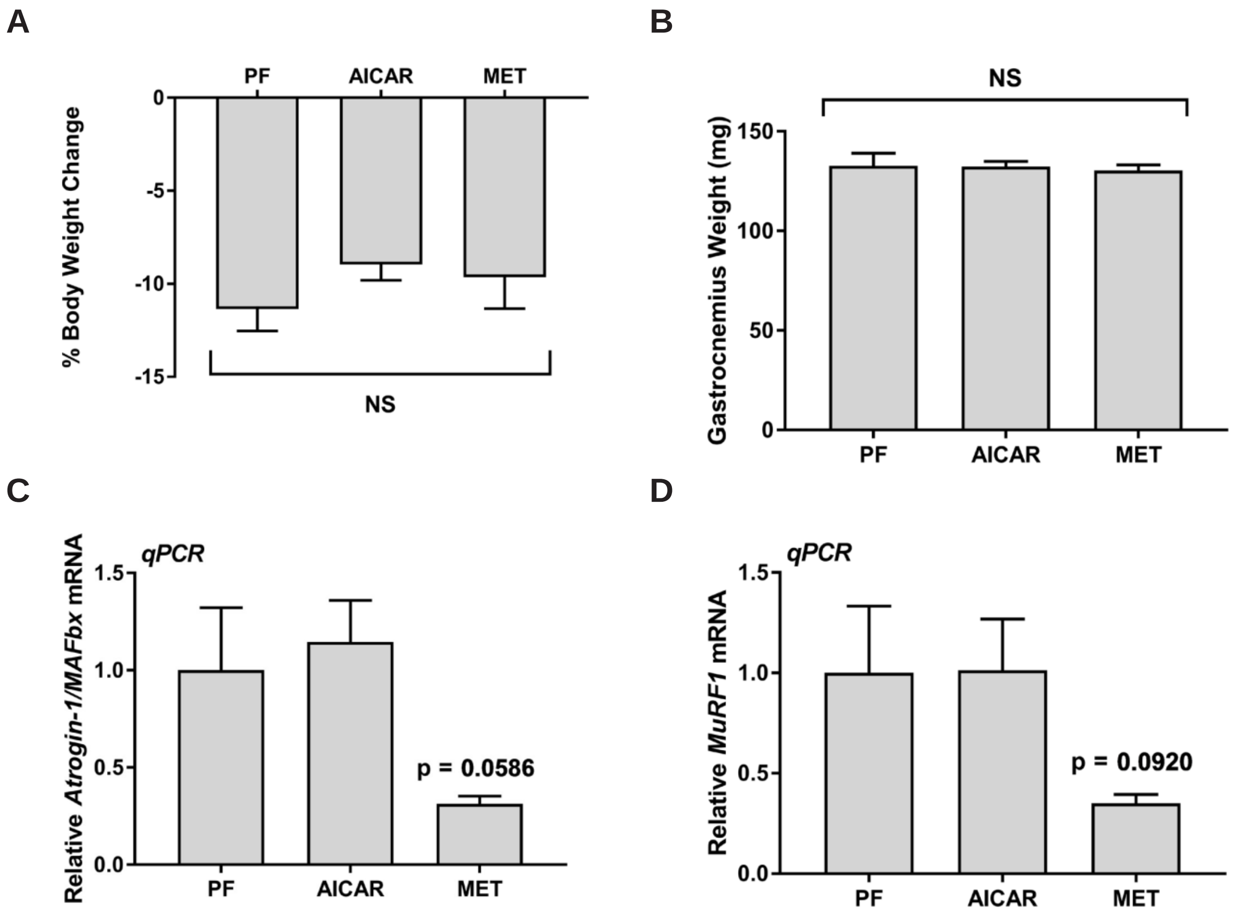

Appendix Figure S3. Single doses of AICAR or metformin alone do not affect body weight or muscle mass.

C57Bl/6 mice were intraperitoneally injected with either AICAR (500mg kg^{-1}), metformin (250mg kg^{-1}), or an equivalent volume of saline. All cohorts were pair-fed (PF) to the food consumption rate of LPS injected mice. Tissue samples were collected 18h later.

(A) Percent body weight change.

(B) Gastrocnemius muscle weights.

(C-D) RT-qPCR analysis of Atrogin-1/MAFbx (C) and MuRF1 (D) mRNA expression from the tibialis anterior.

Data information: Results are derived from four mice per cohort (n = 4) and error bars represent the SEM. Significance between means was first determined using ANOVA. Significance p-values were calculated using Fisher's LSD. NS, not significant.

Appendix Table S1. qPCR Primer Sequences.

Gene	Protein	Gene ID	Forward Primer (5'-3')	Reverse Primer (5'-3')
Nos2	iNOS	18126	GTGCGCATGGCTCGGGATGT	GGCTGTCAGAGCCTCGTGGC
Gapdh	GAPDH	14433	AAGGTCATCCCAGAGCTGAA	AGGAGACAACCTGGTCCTCA
Myod1	MyoD	17927	CGACACCGCCTACTACAGTG	TTCTGTGTCGCTTAGGGATG
Myog	Myogenin	17928	CTACAGGCCTTGCTCAGCTC	AGATTGTGGGCGTCTGTAGG
Fbxo3	Atrogin-1 /MAFbx	67731	GACCGGCTACTGTGGAAGAG	CCAGGAGAGAATGTGGCAGT
Trim63	MuRF1	433766	GAGCAAGGCTTTGAGAACATGGACT	GCGTCCAGAGCGTGTCTCACT

Appendix Table S2. Figure 1 Significance p-values.

Figure	Panel	Control	Treatment	p-value	Symbol
1	A, pAMPK/AMPK	NT	AICAR	0.0026	**
1	A, pAMPK/AMPK	NT	MET	< 0.0001	**
1	A, pAMPK/AMPK	NT	IFNγ/TNFα	< 0.0001	**
1	A, pAMPK/AMPK	NT	IFNγ/TNFα + AICAR	0.0003	**
1	A, pAMPK/AMPK	NT	IFNγ/TNFα + MET	0.0002	**
1	A, pAMPK/AMPK	IFNγ/TNFα	IFNγ/TNFα + AICAR	0.0923	-
1	A, pAMPK/AMPK	IFNγ/TNFα	IFNγ/TNFα + MET	0.1301	-
1	A, pACC/ACC	NT	AICAR	0.0013	**
1	A, pACC/ACC	NT	MET	0.0247	*
1	A, pACC/ACC	NT	IFNγ/TNFα	0.0015	**
1	A, pACC/ACC	NT	IFNγ/TNFα + AICAR	0.0010	**
1	A, pACC/ACC	NT	IFNγ/TNFα + MET	0.0342	*
1	A, pACC/ACC	IFNγ/TNFα	IFNγ/TNFα + AICAR	0.7911	-
1	A, pACC/ACC	IFNγ/TNFα	IFNγ/TNFα + MET	0.1180	-
1	C	NT	AICAR	0.4964	-
1	C	NT	MET	0.0287	*
1	C	NT	IFNγ/TNFα	0.0033	**
1	C	NT	IFNγ/TNFα + AICAR	0.9965	-
1	C	NT	IFNγ/TNFα + MET	0.0009	**
1	C	IFNγ/TNFα	IFNγ/TNFα + AICAR	0.0033	-
1	C	IFNγ/TNFα	IFNγ/TNFα + MET	0.4991	-
1	D	NT	AICAR	0.0047	**
1	D	NT	MET	0.1318	-
1	D	NT	IFNγ/TNFα	< 0.0001	**
1	D	NT	IFNγ/TNFα + AICAR	0.0002	**
1	D	NT	IFNγ/TNFα + MET	< 0.0001	**
1	D	IFNγ/TNFα	IFNγ/TNFα + AICAR	0.0004	††
1	D	IFNγ/TNFα	IFNγ/TNFα + MET	0.9299	-
1	E	NT	AICAR	0.6955	-
1	E	NT	MET	0.9582	-
1	E	NT	IFNγ/TNFα	0.0062	**
1	E	NT	IFNγ/TNFα + AICAR	0.7342	-
1	E	NT	IFNγ/TNFα + MET	0.0059	**
1	E	IFNγ/TNFα	IFNγ/TNFα + AICAR	0.0118	-
1	E	IFNγ/TNFα	IFNγ/TNFα + MET	0.9791	-

Abbreviations: NT, non-treated. MET, metformin.

Appendix Table S3. Figure 2 Significance p-values.

Figure	Panel	Control	Treatment	p-value	Symbol
2	A	NT	AICAR	0.7561	-
2	A	NT	MET	0.0165	*
2	A	NT	IFNγ/TNFα	0.0025	**
2	A	NT	IFNγ/TNFα + AICAR	0.4003	-
2	A	NT	IFNγ/TNFα + MET	0.0005	**
2	A	IFNγ/TNFα	IFNγ/TNFα + AICAR	0.0125	-
2	A	IFNγ/TNFα	IFNγ/TNFα + MET	0.4025	-
2	B	NT	AICAR	0.9267	-
2	B	NT	MET	0.0208	*
2	B	NT	IFNγ/TNFα	0.0025	**
2	B	NT	IFNγ/TNFα + AICAR	0.1200	-
2	B	NT	IFNγ/TNFα + MET	0.0019	**
2	B	IFNγ/TNFα	IFNγ/TNFα + AICAR	0.0549	-
2	B	IFNγ/TNFα	IFNγ/TNFα + MET	0.8694	-

Abbreviations: NT, non-treated. MET, metformin.

Appendix Table S4. Figure 3 Significance p-values.

Figure	Panel	Control	Treatment	p-value	Symbol
3	A, pS6K/S6K	NT	AICAR	0.0954	-
3	A, pS6K/S6K	NT	MET	0.1038	-
3	A, pS6K/S6K	NT	IFNγ/TNFα	<0.0001	**
3	A, pS6K/S6K	NT	IFNγ/TNFα + AICAR	0.0380	*
3	A, pS6K/S6K	NT	IFNγ/TNFα + MET	<0.0001	**
3	A, pS6K/S6K	IFNγ/TNFα	IFNγ/TNFα + AICAR	0.0005	††
3	A, pS6K/S6K	IFNγ/TNFα	IFNγ/TNFα + MET	0.8461	-
3	A, pS6/S6	NT	AICAR	0.8372	-
3	A, pS6/S6	NT	MET	0.8911	-
3	A, pS6/S6	NT	IFNγ/TNFα	0.0372	*
3	A, pS6/S6	NT	IFNγ/TNFα + AICAR	0.7753	-
3	A, pS6/S6	NT	IFNγ/TNFα + MET	0.0435	*
3	A, pS6/S6	IFNγ/TNFα	IFNγ/TNFα + AICAR	0.0205	-
3	A, pS6/S6	IFNγ/TNFα	IFNγ/TNFα + MET	0.9382	-
3	B	NT	AICAR	0.7810	-
3	B	NT	MET	0.1265	-
3	B	NT	IFNγ/TNFα	0.0007	**
3	B	NT	IFNγ/TNFα + AICAR	0.1411	-
3	B	NT	IFNγ/TNFα + MET	0.0001	**
3	B	IFNγ/TNFα	IFNγ/TNFα + AICAR	0.0119	-
3	B	IFNγ/TNFα	IFNγ/TNFα + MET	0.2845	-

Abbreviations: NT, non-treated. MET, metformin.

Appendix Table S5. Figure 4 Significance p-values.

Figure	Panel	Control	Treatment	p-value	Symbol
4	A	IFNγ/TNFα	IFNγ/TNFα + AICAR	0.0005	††
4	A	IFNγ/TNFα	IFNγ/TNFα + MET	0.3321	-
4	B	IFNγ/TNFα	IFNγ/TNFα + AICAR	0.0014	††
4	B	IFNγ/TNFα	IFNγ/TNFα + MET	0.3702	-
4	C	IFNγ/TNFα	IFNγ/TNFα + AICAR	<0.0001	††
4	C	IFNγ/TNFα	IFNγ/TNFα + MET	0.1597	-
4	D	siCTL<br>+ IFNγ/TNFα<br>+ AICAR 0.5mM	siAMPK<br>+ IFNγ/TNFα<br>+ AICAR 0.5mM	0.0071	**
4	D	siCTL<br>+ IFNγ/TNFα<br>+ AICAR 1.0mM	siAMPK<br>+ IFNγ/TNFα<br>+ AICAR 1.0mM	0.0206	*

Abbreviations: MET, metformin.

Appendix Table S6. Figure 5 Significance p-values.

Figure	Panel	Control	Treatment	p-value	Symbol
5	A, pAMPK/AMPK	NT	AICAR	0.0203	*
5	A, pAMPK/AMPK	NT	CC	0.9541	-
5	A, pAMPK/AMPK	NT	CC + AICAR	0.0217	*
5	A, pACC/ACC	NT	AICAR	0.0010	**
5	A, pACC/ACC	NT	CC	0.5689	-
5	A, pACC/ACC	NT	CC + AICAR	0.2597	-
5	B	NT	AICAR	0.0264	*
5	B	NT	IFNγ/TNFα	0.0008	**
5	B	NT	IFNγ/TNFα + AICAR	0.0672	-
5	B	NT	CC	0.1400	-
5	B	NT	CC + AICAR	0.1231	-
5	B	NT	CC + IFNγ/TNFα	0.0168	*
5	B	NT	CC + IFNγ/TNFα + AICAR	0.0331	*
5	B	IFNγ/TNFα	IFNγ/TNFα + AICAR	<0.0001	-
5	B	IFNγ/TNFα	CC + IFNγ/TNFα	0.1689	-
5	B	IFNγ/TNFα	CC + IFNγ/TNFα + AICAR	0.0940	-
5	C	NT	AICAR	<0.0001	**
5	C	NT	IFNγ/TNFα	<0.0001	**
5	C	NT	IFNγ/TNFα + AICAR	0.3061	-
5	C	NT	CC	<0.0001	**
5	C	NT	CC + AICAR	<0.0001	**
5	C	NT	CC + IFNγ/TNFα	<0.0001	**
5	C	NT	CC + IFNγ/TNFα + AICAR	<0.0001	**
5	C	IFNγ/TNFα	IFNγ/TNFα + AICAR	<0.0001	-
5	C	IFNγ/TNFα	CC + IFNγ/TNFα	0.7305	-
5	C	IFNγ/TNFα	CC + IFNγ/TNFα + AICAR	<0.0001	-
5	D	NT	AICAR	0.2850	-
5	D	NT	IFNγ/TNFα	<0.0001	**
5	D	NT	IFNγ/TNFα + AICAR	0.0032	**
5	D	NT	CC	0.3247	-
5	D	NT	CC + AICAR	0.4981	-
5	D	NT	CC + IFNγ/TNFα	<0.0001	**
5	D	NT	CC + IFNγ/TNFα + AICAR	0.0002	**
5	D	IFNγ/TNFα	IFNγ/TNFα + AICAR	0.0202	†
5	D	IFNγ/TNFα	CC + IFNγ/TNFα	0.9443	-
5	D	IFNγ/TNFα	CC + IFNγ/TNFα + AICAR	0.1561	-
5	E, pS6K/S6K	NT	AICAR	0.1220	-

5	E, pS6K/S6K	NT	IFNγ/TNFα	<0.0001	**
5	E, pS6K/S6K	NT	IFNγ/TNFα + AICAR	0.8872	-
5	E, pS6K/S6K	NT	CC	0.0073	**
5	E, pS6K/S6K	NT	CC + AICAR	<0.0001	**
5	E, pS6K/S6K	NT	CC + IFNγ/TNFα	<0.0001	**
5	E, pS6K/S6K	NT	CC + IFNγ/TNFα + AICAR	<0.0001	**
5	E, pS6K/S6K	IFNγ/TNFα	IFNγ/TNFα + AICAR	<0.0001	-
5	E, pS6K/S6K	IFNγ/TNFα	CC + IFNγ/TNFα	0.9411	-
5	E, pS6K/S6K	IFNγ/TNFα	CC + IFNγ/TNFα + AICAR	0.9537	-
5	E, pS6/S6	NT	AICAR	0.1713	-
5	E, pS6/S6	NT	IFNγ/TNFα	0.0065	**
5	E, pS6/S6	NT	IFNγ/TNFα + AICAR	0.8046	-
5	E, pS6/S6	NT	CC	0.4632	-
5	E, pS6/S6	NT	CC + AICAR	0.2567	-
5	E, pS6/S6	NT	CC + IFNγ/TNFα	0.0067	**
5	E, pS6/S6	NT	CC + IFNγ/TNFα + AICAR	0.0064	**
5	E, pS6/S6	IFNγ/TNFα	IFNγ/TNFα + AICAR	0.0038	-
5	E, pS6/S6	IFNγ/TNFα	CC + IFNγ/TNFα	0.9881	-
5	E, pS6/S6	IFNγ/TNFα	CC + IFNγ/TNFα + AICAR	0.9945	-
5	F	IFNγ/TNFα	IFNγ/TNFα + AICAR	0.0103	†
5	F	IFNγ/TNFα	CC + IFNγ/TNFα	0.6912	-
5	F	IFNγ/TNFα	CC + IFNγ/TNFα + AICAR	0.4240	-

Abbreviations: NT, non-treated. CC, Compound C

Appendix Table S7. Figure 6 Significance p-values

Figure	Panel	Control	Treatment	p-value	Symbol
6	A	DMSO	A-769662	0.1967	-
6	A	DMSO	IFNγ/TNFα + DMSO	0.0029	**
6	A	DMSO	IFNγ/TNFα + A-769662	0.6624	-
6	A	IFNγ/TNFα + DMSO	IFNγ/TNFα + A-769662	0.0016	-
6	B	DMSO	A-769662	0.0011	**
6	B	DMSO	IFNγ/TNFα + DMSO	<0.0001	**
6	B	DMSO	IFNγ/TNFα + A-769662	0.1628	-
6	B	IFNγ/TNFα + DMSO	IFNγ/TNFα + A-769662	<0.0001	-
6	C	DMSO	A-769662	0.0815	-
6	C	DMSO	IFNγ/TNFα + DMSO	0.0031	**
6	C	DMSO	IFNγ/TNFα + A-769662	>0.9999	-
6	C	IFNγ/TNFα + DMSO	IFNγ/TNFα + A-769662	0.0031	-
6	D, pS6K/S6K	DMSO	A-769662	0.9780	-
6	D, pS6K/S6K	DMSO	IFNγ/TNFα + DMSO	0.0060	**
6	D, pS6K/S6K	DMSO	IFNγ/TNFα + A-769662	0.0501	-
6	D, pS6K/S6K	IFNγ/TNFα + DMSO	IFNγ/TNFα + A-769662	0.0003	-
6	D, pS6/S6	DMSO	A-769662	0.5221	-
6	D, pS6/S6	DMSO	IFNγ/TNFα + DMSO	0.0011	**
6	D, pS6/S6	DMSO	IFNγ/TNFα + A-769662	0.0650	-
6	D, pS6/S6	IFNγ/TNFα + DMSO	IFNγ/TNFα + A-769662	0.0221	-
6	E	IFNγ/TNFα + DMSO	IFNγ/TNFα + A-769662	0.0002	††
6	F	IFNγ/TNFα + DMSO	IFNγ/TNFα + A-769662	0.0001	††
6	G	IFNγ/TNFα + DMSO	IFNγ/TNFα + A-769662	<0.0001	††

Appendix Table S8. Figure 7 Significance p-values

Figure	Panel	Control	Treatment	p-value	Symbol
7	A	DMSO	A-769662 25μM	0.7863	-
7	A	DMSO	A-769662 50μM	0.4685	-
7	A	DMSO	IFNγ/TNFα<br>+ DMSO	<0.0001	**
7	A	DMSO	IFNγ/TNFα<br>+ A-769662 25μM	0.0004	**
7	A	DMSO	IFNγ/TNFα<br>+ A-769662 50μM	0.0002	**
7	A	DMSO	IFNγ/TNFα + AICAR 125μM<br>+ DMSO	0.0002	**
7	A	DMSO	IFNγ/TNFα + AICAR 125μM<br>+ A-769662 25μM	0.0010	**
7	A	DMSO	IFNγ/TNFα + AICAR 125μM<br>+ A-769662 50μM	0.0034	**
7	A	DMSO	IFNγ/TNFα + AICAR 250μM<br>+ DMSO	0.0006	**
7	A	DMSO	IFNγ/TNFα + AICAR 250μM<br>+ A-769662 25μM	0.0647	-
7	A	DMSO	IFNγ/TNFα + AICAR 250μM<br>+ A-769662 50μM	0.8389	-
7	A	IFNγ/TNFα<br>+ DMSO	IFNγ/TNFα<br>+ A-769662 25μM	0.2053	-
7	A	IFNγ/TNFα<br>+ DMSO	IFNγ/TNFα<br>+ A-769662 50μM	0.3064	-
7	A	IFNγ/TNFα<br>+ DMSO	IFNγ/TNFα + AICAR 125μM<br>+ DMSO	0.3386	-
7	A	IFNγ/TNFα<br>+ DMSO	IFNγ/TNFα + AICAR 125μM<br>+ A-769662 25μM	0.1028	-
7	A	IFNγ/TNFα<br>+ DMSO	IFNγ/TNFα + AICAR 125μM<br>+ A-769662 50μM	0.0382	†
7	A	IFNγ/TNFα<br>+ DMSO	IFNγ/TNFα + AICAR 250μM<br>+ DMSO	0.1491	-
7	A	IFNγ/TNFα<br>+ DMSO	IFNγ/TNFα + AICAR 250μM<br>+ A-769662 25μM	0.0018	-
7	A	IFNγ/TNFα<br>+ DMSO	IFNγ/TNFα + AICAR 250μM<br>+ A-769662 50μM	<0.0001	-
7	B	IFNγ/TNFα<br>+ DMSO	IFNγ/TNFα<br>+ A-769662 25μM	0.4926	-
7	B	IFNγ/TNFα<br>+ DMSO	IFNγ/TNFα<br>+ A-769662 50μM	0.2623	-
7	B	IFNγ/TNFα<br>+ DMSO	IFNγ/TNFα + AICAR 125μM<br>+ DMSO	0.0797	-

7	B	IFNγ/TNFα + DMSO	IFNγ/TNFα + AICAR 125μM + A-769662 25μM	0.6749	-
7	B	IFNγ/TNFα + DMSO	IFNγ/TNFα + AICAR 125μM + A-769662 50μM	0.0025	††
7	B	IFNγ/TNFα + DMSO	IFNγ/TNFα + AICAR 250μM + DMSO	<0.0001	††
7	B	IFNγ/TNFα + DMSO	IFNγ/TNFα + AICAR 250μM + A-769662 25μM	<0.0001	††
7	B	IFNγ/TNFα + DMSO	IFNγ/TNFα + AICAR 250μM + A-769662 50μM	<0.0001	††
7	C, pS6K/S6K	DMSO	A-769662 25μM	0.0325	*
7	C, pS6K/S6K	DMSO	A-769662 50μM	0.0661	-
7	C, pS6K/S6K	DMSO	IFNγ/TNFα + DMSO	<0.0001	**
7	C, pS6K/S6K	DMSO	IFNγ/TNFα + A-769662 25μM	<0.0001	**
7	C, pS6K/S6K	DMSO	IFNγ/TNFα + A-769662 50μM	<0.0001	**
7	C, pS6K/S6K	DMSO	IFNγ/TNFα + AICAR 125μM + DMSO	<0.0001	**
7	C, pS6K/S6K	DMSO	IFNγ/TNFα + AICAR 125μM + A-769662 25μM	<0.0001	**
7	C, pS6K/S6K	DMSO	IFNγ/TNFα + AICAR 125μM + A-769662 50μM	<0.0001	**
7	C, pS6K/S6K	DMSO	IFNγ/TNFα + AICAR 250μM + DMSO	<0.0001	**
7	C, pS6K/S6K	DMSO	IFNγ/TNFα + AICAR 250μM + A-769662 25μM	0.0069	**
7	C, pS6K/S6K	DMSO	IFNγ/TNFα + AICAR 250μM + A-769662 50μM	0.0809	-
7	C, pS6K/S6K	IFNγ/TNFα + DMSO	IFNγ/TNFα + A-769662 25μM	0.7856	-
7	C, pS6K/S6K	IFNγ/TNFα + DMSO	IFNγ/TNFα + A-769662 50μM	0.7596	-
7	C, pS6K/S6K	IFNγ/TNFα + DMSO	IFNγ/TNFα + AICAR 125μM + DMSO	0.7596	-
7	C, pS6K/S6K	IFNγ/TNFα + DMSO	IFNγ/TNFα + AICAR 125μM + A-769662 25μM	0.8918	-
7	C, pS6K/S6K	IFNγ/TNFα + DMSO	IFNγ/TNFα + AICAR 125μM + A-769662 50μM	0.0039	††
7	C, pS6K/S6K	IFNγ/TNFα + DMSO	IFNγ/TNFα + AICAR 250μM + DMSO	0.0350	†

7	C, pS6K/S6K	IFNγ/TNFα + DMSO	IFNγ/TNFα + AICAR 250μM + A-769662 25μM	<0.0001	††
7	C, pS6K/S6K	IFNγ/TNFα + DMSO	IFNγ/TNFα + AICAR 250μM + A-769662 50μM	<0.0001	-
7	C, pS6/S6	DMSO	A-769662 25μM	0.7896	-
7	C, pS6/S6	DMSO	A-769662 50μM	0.6209	-
7	C, pS6/S6	DMSO	IFNγ/TNFα + DMSO	<0.0001	**
7	C, pS6/S6	DMSO	IFNγ/TNFα + A-769662 25μM	<0.0001	**
7	C, pS6/S6	DMSO	IFNγ/TNFα + A-769662 50μM	<0.0001	**
7	C, pS6/S6	DMSO	IFNγ/TNFα + AICAR 125μM + DMSO	<0.0001	**
7	C, pS6/S6	DMSO	IFNγ/TNFα + AICAR 125μM + A-769662 25μM	<0.0001	**
7	C, pS6/S6	DMSO	IFNγ/TNFα + AICAR 125μM + A-769662 50μM	0.0004	**
7	C, pS6/S6	DMSO	IFNγ/TNFα + AICAR 250μM + DMSO	<0.0001	**
7	C, pS6/S6	DMSO	IFNγ/TNFα + AICAR 250μM + A-769662 25μM	0.0017	**
7	C, pS6/S6	DMSO	IFNγ/TNFα + AICAR 250μM + A-769662 50μM	0.0994	-
7	C, pS6/S6	IFNγ/TNFα + DMSO	IFNγ/TNFα + A-769662 25μM	0.7981	-
7	C, pS6/S6	IFNγ/TNFα + DMSO	IFNγ/TNFα + A-769662 50μM	0.9773	-
7	C, pS6/S6	IFNγ/TNFα + DMSO	IFNγ/TNFα + AICAR 125μM + DMSO	0.9094	-
7	C, pS6/S6	IFNγ/TNFα + DMSO	IFNγ/TNFα + AICAR 125μM + A-769662 25μM	0.4134	-
7	C, pS6/S6	IFNγ/TNFα + DMSO	IFNγ/TNFα + AICAR 125μM + A-769662 50μM	0.0012	††
7	C, pS6/S6	IFNγ/TNFα + DMSO	IFNγ/TNFα + AICAR 250μM + DMSO	0.3984	-
7	C, pS6/S6	IFNγ/TNFα + DMSO	IFNγ/TNFα + AICAR 250μM + A-769662 25μM	0.0021	††
7	C, pS6/S6	IFNγ/TNFα + DMSO	IFNγ/TNFα + AICAR 250μM + A-769662 50μM	<0.0001	-

Appendix Table S9. Figure 8 Significance p-values

Figure	Panel	Control	Treatment	p-value	Symbol
8	C	Saline	C26	<0.0001	**
8	C	Saline	C26 + AICAR	<0.0001	**
8	C	Saline	C26 + MET	<0.0001	**
8	C	C26	C26 + AICAR	<0.0001	††
8	C	C26	C26 + MET	0.0194	†
8	D	Saline	C26	0.0043	**
8	D	Saline	C26 + AICAR	0.0956	-
8	D	Saline	C26 + MET	0.0081	**
8	D	C26	C26 + AICAR	0.0743	-
8	D	C26	C26 + MET	0.6698	-
8	E	Saline	C26	0.0190	*
8	E	Saline	C26 + AICAR	0.0690	-
8	E	Saline	C26 + MET	0.0268	*
8	E	C26	C26 + AICAR	0.4305	-
8	E	C26	C26 + MET	0.8296	-

Abbreviations: MET, metformin.

Appendix Table S10. Figure 9 Significance p-values

Figure	Panel	Control	Treatment	p-value	Symbol
9	A	PF	LPS	<0.0001	**
9	A	PF	LPS + AICAR	0.1205	-
9	A	PF	LPS + MET	<0.0001	**
9	A	LPS	LPS + AICAR	<0.0001	-
9	A	LPS	LPS + MET	0.9987	-
9	B	PF	LPS	0.0194	*
9	B	PF	LPS + AICAR	0.5364	-
9	B	PF	LPS + MET	0.0233	*
9	B	LPS	LPS + AICAR	0.0670	-
9	B	LPS	LPS + MET	0.9297	-
9	C	PF	LPS	0.0026	**
9	C	PF	LPS + AICAR	0.1666	-
9	C	PF	LPS + MET	0.0040	**
9	C	LPS	LPS + AICAR	0.0506	-
9	C	LPS	LPS + MET	0.8428	-

Abbreviations: PF, pair-fed controls. MET, metformin.

Appendix Table S11. Figure EV3 Significance p-values

Figure	Panel	Control	Treatment	p-value	Symbol
EV3	A	C26	C26 + AICAR	0.0113	†
EV3	A	C26	C26 + MET	0.0180	†
EV3	B	Saline	C26	0.0078	**
EV3	B	Saline	C26 + AICAR	0.7341	-
EV3	B	Saline	C26 + MET	0.7652	-
EV3	B	C26	C26 + AICAR	0.0047	-
EV3	B	C26	C26 + MET	0.0050	-

Abbreviations: MET, metformin.

Appendix Table S12. Figure EV4 Significance p-values

Figure	Panel	Control	Treatment	p-value	Symbol
EV4	B	Day 14, Saline	Day 14, C26	0.0107	*
EV4	B	Day 14, Saline	Day 14, C26 + AICAR	0.0049	**
EV4	B	Day 21, Saline	Day 21, C26	<0.0001	**
EV4	B	Day 21, Saline	Day 21, C26 + AICAR	0.0004	**
EV4	B	Day 14, Saline	Day 21, Saline	0.9795	-
EV4	B	Day 14, C26	Day 21, C26	0.0001	**
EV4	B	Day 14, C26 + AICAR	Day 21, C26 + AICAR	0.0889	NS
EV4	C, Atrogin1	Saline	C26	0.0003	**
EV4	C, Atrogin1	Saline	C26 + AICAR	0.3442	-
EV4	C, Atrogin1	C26	C26 + AICAR	0.0012	-
EV4	C, MuRF1	Saline	C26	0.0074	**
EV4	C, MuRF1	Saline	C26 + AICAR	0.0173	*
EV4	C, MuRF1	C26	C26 + AICAR	0.6088	-

Abbreviations: NS, not significant

Appendix Table S13. Figure EV5 Significance p-values

Figure	Panel	Control	Treatment	p-value	Symbol
EV5	A	Saline	AICAR	0.6095	-
EV5	A	Saline	IFNγ/TNFα	0.0010	**
EV5	A	Saline	IFNγ/TNFα + AICAR	0.0010	**
EV5	A	IFNγ/TNFα	IFNγ/TNFα + AICAR	0.9099	-
EV5	B	Saline	AICAR	0.4364	-
EV5	B	Saline	IFNγ/TNFα	0.0244	*
EV5	B	Saline	IFNγ/TNFα + AICAR	0.7582	NS
EV5	B	IFNγ/TNFα	IFNγ/TNFα + AICAR	0.0554	-

Abbreviations: NS, not significant

Appendix Table S14. Table 1 Significance p-values

Table	Variable	Control	Treatment	p-value	Symbol
1	Body Weight Change	Saline	C26	<0.0001	**
1	Body Weight Change	Saline	C26 + AICAR	<0.0001	**
1	Body Weight Change	Saline	C26 + MET	<0.0001	**
1	Body Weight Change	C26	C26 + AICAR	0.0004	††
1	Body Weight Change	C26	C26 + MET	0.2284	-
1	Tibialis Anterior	Saline	C26	<0.0001	**
1	Tibialis Anterior	Saline	C26 + AICAR	0.0160	*
1	Tibialis Anterior	Saline	C26 + MET	<0.0001	**
1	Tibialis Anterior	C26	C26 + AICAR	0.0341	†
1	Tibialis Anterior	C26	C26 + MET	0.7884	-
1	Gastrocnemius	Saline	C26	<0.0001	**
1	Gastrocnemius	Saline	C26 + AICAR	0.0006	**
1	Gastrocnemius	Saline	C26 + MET	<0.0001	**
1	Gastrocnemius	C26	C26 + AICAR	0.0107	†
1	Gastrocnemius	C26	C26 + MET	0.4784	-
1	Fat Pad	Saline	C26	<0.0001	**
1	Fat Pad	Saline	C26 + AICAR	<0.0001	**
1	Fat Pad	Saline	C26 + MET	<0.0001	**
1	Fat Pad	C26	C26 + AICAR	0.6562	-
1	Fat Pad	C26	C26 + MET	0.9207	-
1	Tumor	C26	C26 + AICAR	0.8997	-
1	Tumor	C26	C26 + MET	0.8605	-
1	IL-6	Saline	C26	0.0019	**
1	IL-6	Saline	C26 + AICAR	0.0105	*
1	IL-6	Saline	C26 + MET	0.1123	-
1	IL-6	C26	C26 + AICAR	0.3767	-
1	IL-6	C26	C26 + MET	0.0455	†

Abbreviations: MET, metformin.

Appendix Table S15. Table 2 Significance p-values

Table	Variable	Control	Treatment	p-value	Symbol
2	Body Weight Change	PF	Veh.	<0.0001	**
2	Body Weight Change	PF	LPS	0.4611	-
2	Body Weight Change	PF	LPS + AICAR	0.0221	*
2	Body Weight Change	PF	LPS + MET	0.1424	-
2	Body Weight Change	LPS	LPS + AICAR	0.0032	-
2	Body Weight Change	LPS	LPS + MET	0.4569	-
2	Tibialis Anterior	PF	Veh.	0.6981	-
2	Tibialis Anterior	PF	LPS	0.0080	**
2	Tibialis Anterior	PF	LPS + AICAR	0.5768	-
2	Tibialis Anterior	PF	LPS + MET	0.0268	*
2	Tibialis Anterior	LPS	LPS + AICAR	0.0316	-
2	Tibialis Anterior	LPS	LPS + MET	0.6255	-
2	Gastrocnemius	PF	Veh.	0.0740	-
2	Gastrocnemius	PF	LPS	0.0330	*
2	Gastrocnemius	PF	LPS + AICAR	0.8263	-
2	Gastrocnemius	PF	LPS + MET	0.0368	*
2	Gastrocnemius	LPS	LPS + AICAR	0.0539	-
2	Gastrocnemius	LPS	LPS + MET	0.9629	-
2	Fat Pad	PF	Veh.	0.9299	-
2	Fat Pad	PF	LPS	0.6192	-
2	Fat Pad	PF	LPS + AICAR	0.9013	-
2	Fat Pad	PF	LPS + MET	0.5519	-
2	Fat Pad	LPS	LPS + AICAR	0.5351	-
2	Fat Pad	LPS	LPS + MET	0.9215	-

Abbreviations: PF, pair-fed controls. Veh., Vehicle. MET, metformin.
