# Supplementary material for: The AMPK agonist 5‐aminoimidazole‐4‐carboxamide ribonucleotide (AICAR), but not metformin, prevents inflammation‐associated cachectic muscle wasting
Source: EMBO Mol Med. 2018 May 29;10(7):e8307. doi: 10.15252/emmm.201708307 (PMC6034131; doi:10.15252/emmm.201708307)
Supplement: Supplementary file 2 — Expanded View Figures PDF [file EMMM-10-e8307-s002.pdf]

Expanded View Figures

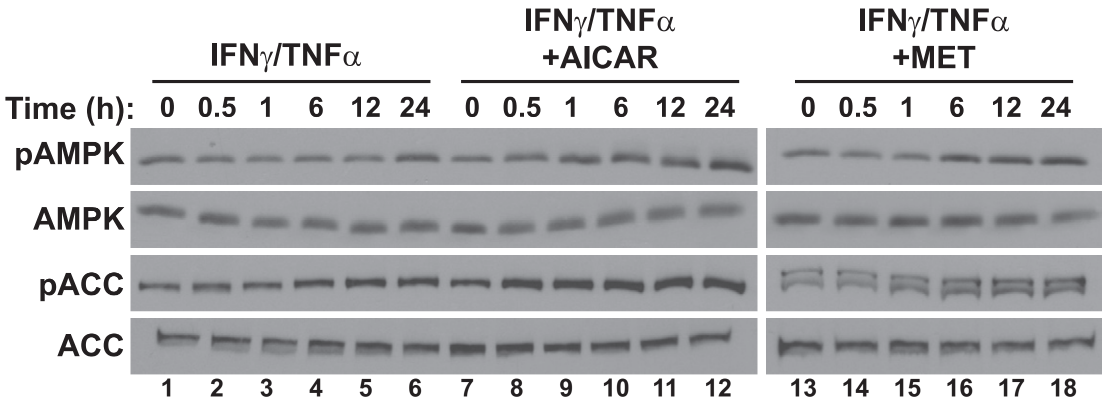

Figure EV1. Time course of AMPK activation during cytokine and AICAR or metformin co-treatment.

Western blotting for phospho-Thr172 AMPKα (pAMPK), total AMPKα (AMPK), phospho-Ser79-ACC (pACC), and total ACC during the first 24 h of treatment.

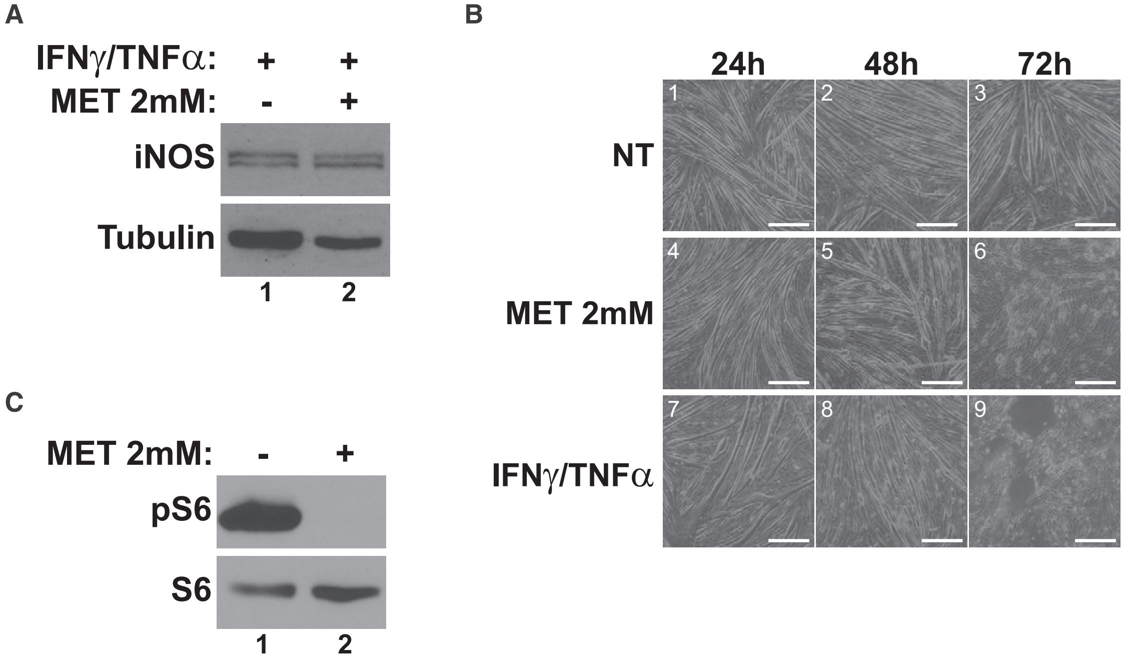

Figure EV2. Higher doses of metformin induce atrophy and do not inhibit iNOS.

A Western blotting for iNOS and tubulin protein levels in cells treated with IFNγ/TNFα with or without metformin (2 mM) for 24 h.
B Phase contrast images at 24 h, 48 h, and 72 h post-treatment with either metformin or IFNγ/TNFα. Scale bars represent 0.25 mm.
C Western blotting of phospho-Ser235/236-S6 (pS6) and total S6 48 h after treatment with metformin.

 © 2018 The Authors

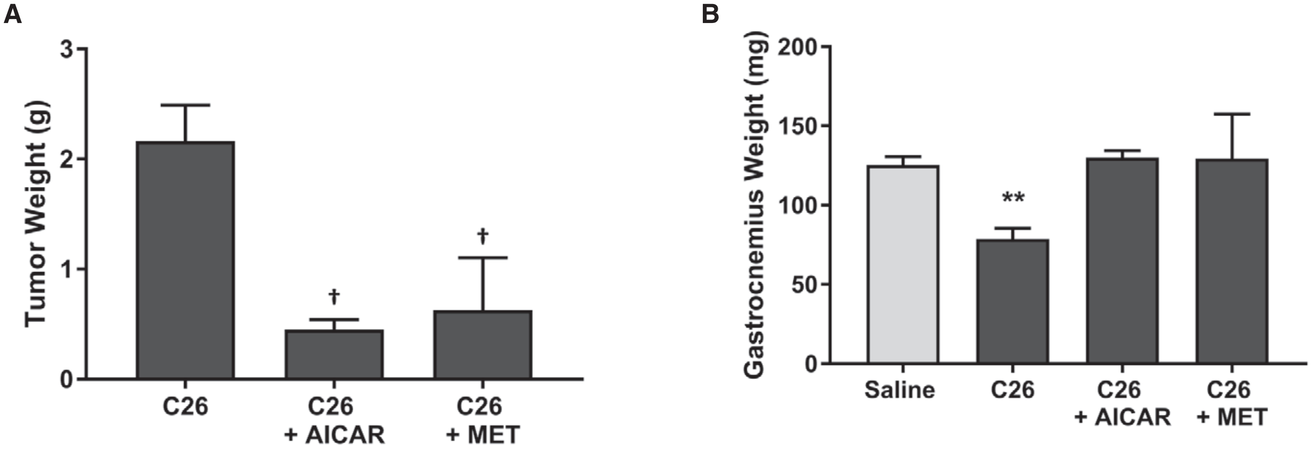

Figure EV3. Early treatment with AMPK agonists impairs tumor development in the C26 model.

Starting 9 days post-C26 cell subcutaneous injection, mice were intraperitoneally injected daily with either AICAR (500 mg/kg/day), metformin (250 mg/kg/day), or an equivalent volume of saline.

A Final tumor masses.
B Gastrocnemius muscle weights.

Data information: Results are derived from three mice per cohort ($n = 3$), and error bars represent the SEM. Significance between means was first determined using ANOVA. Significance P-values were calculated using Fisher's LSD. $^{**}P < 0.01$ from saline controls; $^{\dagger}P < 0.05$ from C26 controls.

© 2018 The Authors

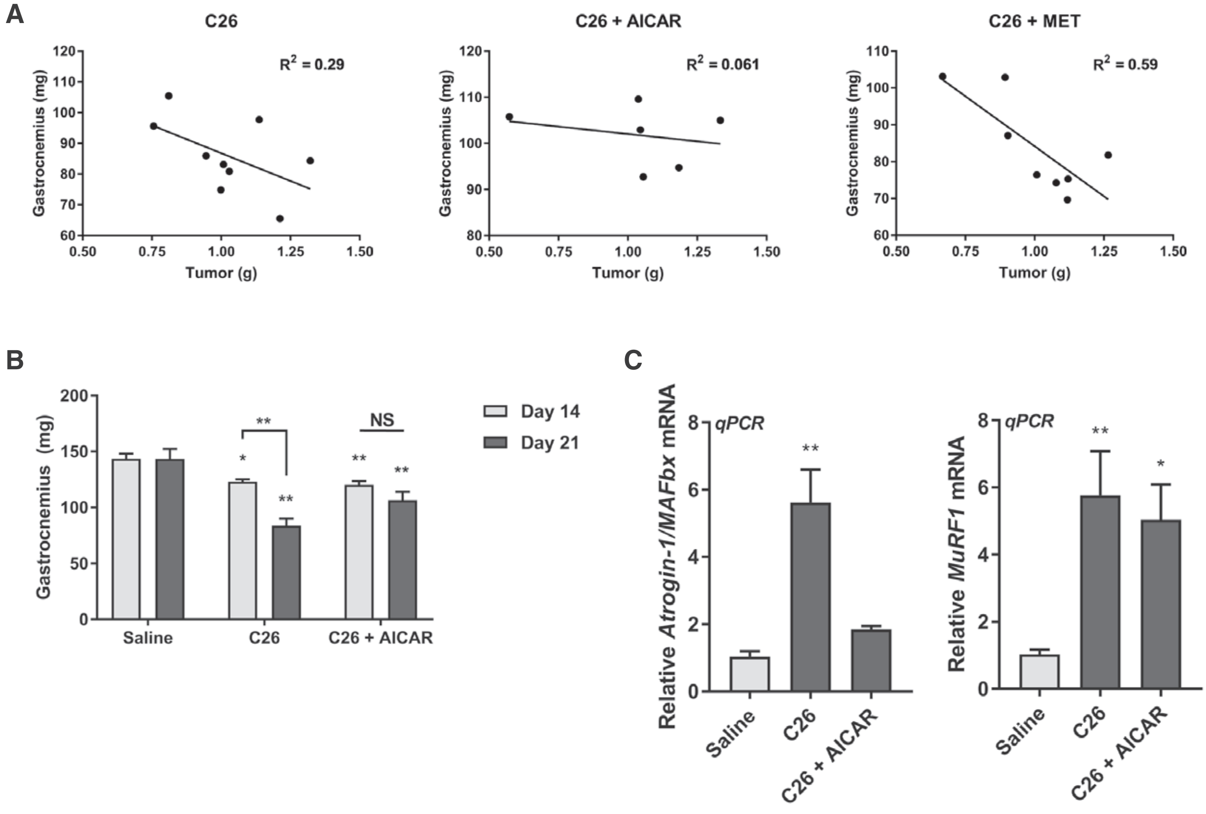

Figure EV4. Delayed AICAR treatment prevents the further progression of cancer-related muscle atrophy.

A Correlation analysis between final tumor mass and gastrocnemius muscle mass in the C26 inoculated cohorts from Table 1.

B, C BALB/C mice were intraperitoneally injected daily with either AICAR (500 mg/kg/day), metformin (250 mg/kg/day), or an equivalent volume of saline starting 12 days after C26 inoculation. Tissues were collected from mice either two days later (Day 14) or nine days later (Day 21). (B) Gastrocnemius muscle weights. (C) RT–qPCR analysis of Atrogin-1/MAFbx and MuRF1 mRNA expression from the tibialis anterior at Day 14.

Data information: Results are derived from four mice per cohort for Day 14 (n = 4) and three mice per cohort for Day 21 (n = 3), and error bars represent the SEM. Significance between means was first determined using ANOVA. Significance P-values were calculated using Fisher's LSD. *$P < 0.05$; **$P < 0.01$ from saline controls or indicated comparisons.

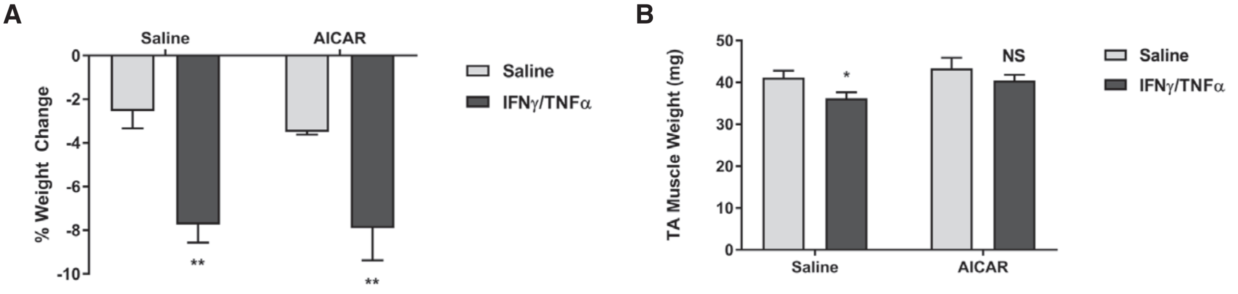

Figure EV5. AICAR treatment prevents muscle wasting induced by intramuscular cytokine injection.

C57Bl/6 mice were intramuscularly injected with either IFNγ (7,500 U) and TNFα (3 μg) or an equivalent volume of saline in the right posterior thigh muscles daily for 5 days. Mice were also intraperitoneally injected with either AICAR (350 mg/kg/day) or an equivalent volume of saline. On the fifth day, mice were euthanized and tissue samples were collected.

A, B Body weight (A) and tibialis anterior muscle weight (B) analysis of saline (n = 8), IFNγ/TNFα (n = 8), AICAR (n = 3), and IFNγ/TNFα + AICAR (n = 7) cohorts.

Data information: Error bars represent the SEM. Significance between means was first determined using ANOVA. Significance P-values were calculated using Fisher's LSD. *$P < 0.05$; **$P < 0.01$ from saline controls. NS, not significant.

© 2018 The Authors
