# Supplementary material for: The AMPK agonist 5‐aminoimidazole‐4‐carboxamide ribonucleotide (AICAR), but not metformin, prevents inflammation‐associated cachectic muscle wasting
Source: EMBO Mol Med. 2018 May 29;10(7):e8307. doi: 10.15252/emmm.201708307 (PMC6034131; doi:10.15252/emmm.201708307)

Figure 1 - Panel A

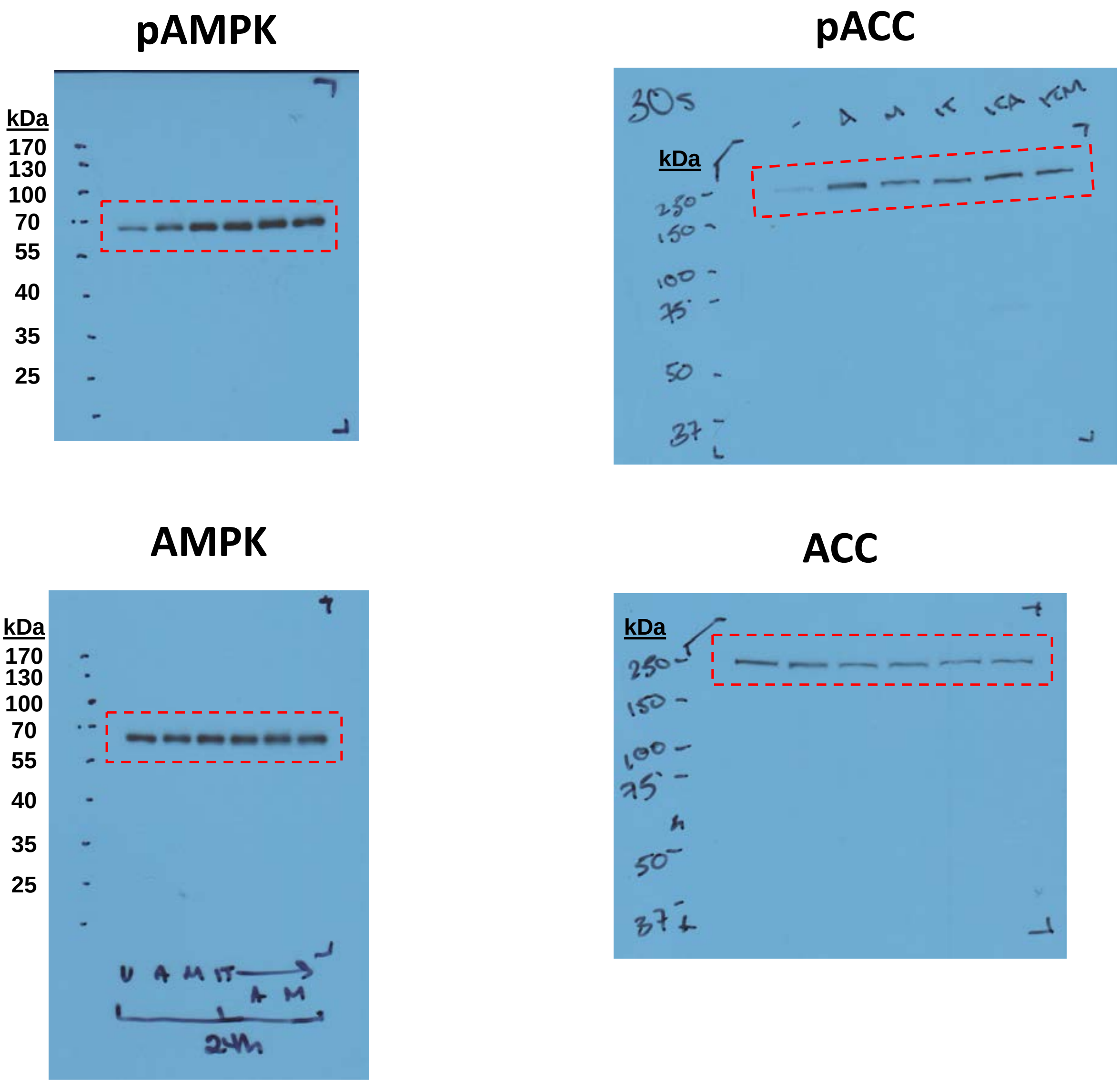

Abbreviations: U or “-”, non-treated. A, AICAR. M, metformin. IT, IFNγ/TNFα

Figure 1 - Panel B

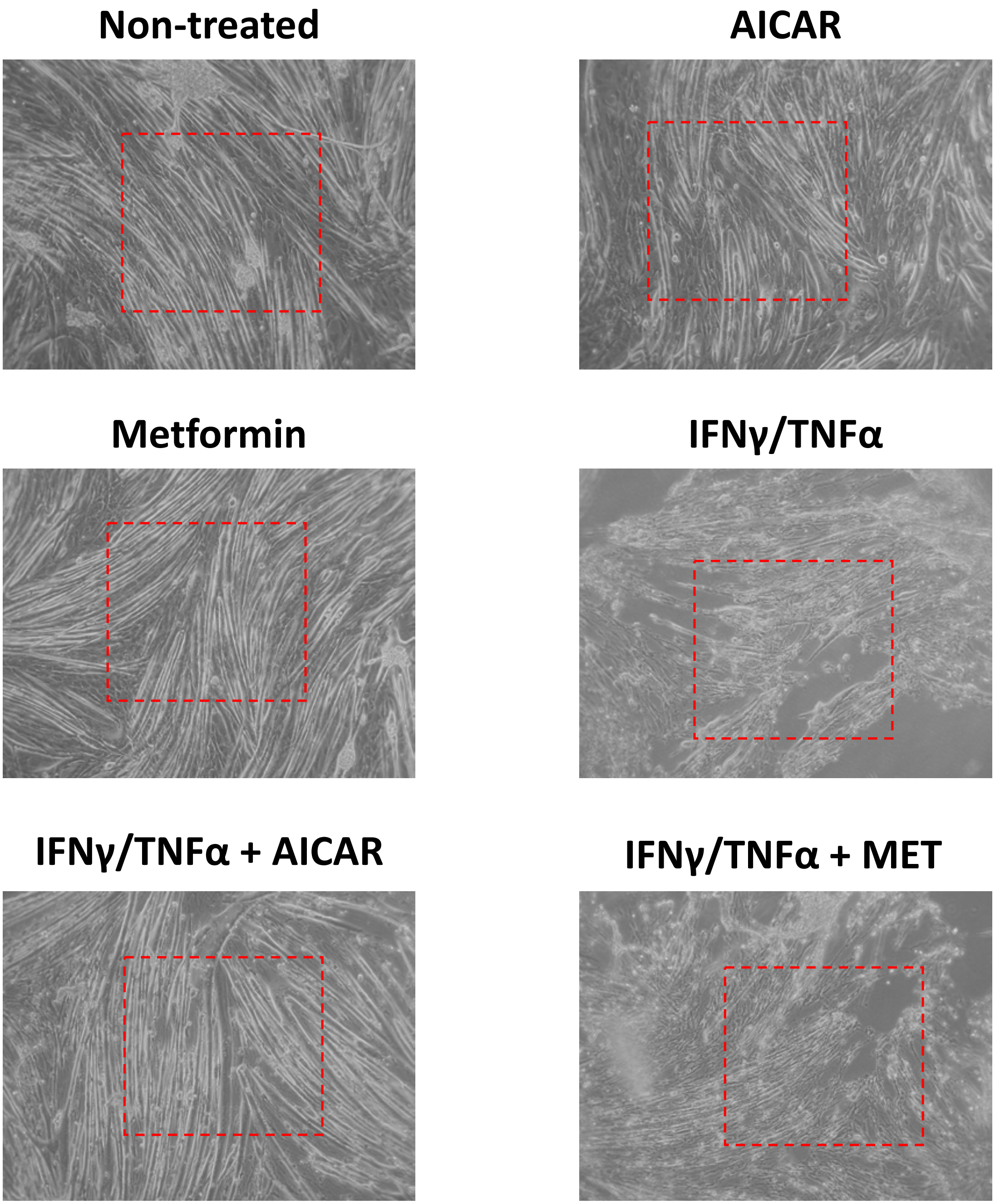

Supplement: Supplementary file 4 — Source Data for Figure 1 [file EMMM-10-e8307-s003.pdf]
