# Supplementary material for: The AMPK agonist 5‐aminoimidazole‐4‐carboxamide ribonucleotide (AICAR), but not metformin, prevents inflammation‐associated cachectic muscle wasting
Source: EMBO Mol Med. 2018 May 29;10(7):e8307. doi: 10.15252/emmm.201708307 (PMC6034131; doi:10.15252/emmm.201708307)
Supplement: Supplementary file 8 — Source Data for Figure 6 [file EMMM-10-e8307-s007.pdf]

Figure 6 - Panel D

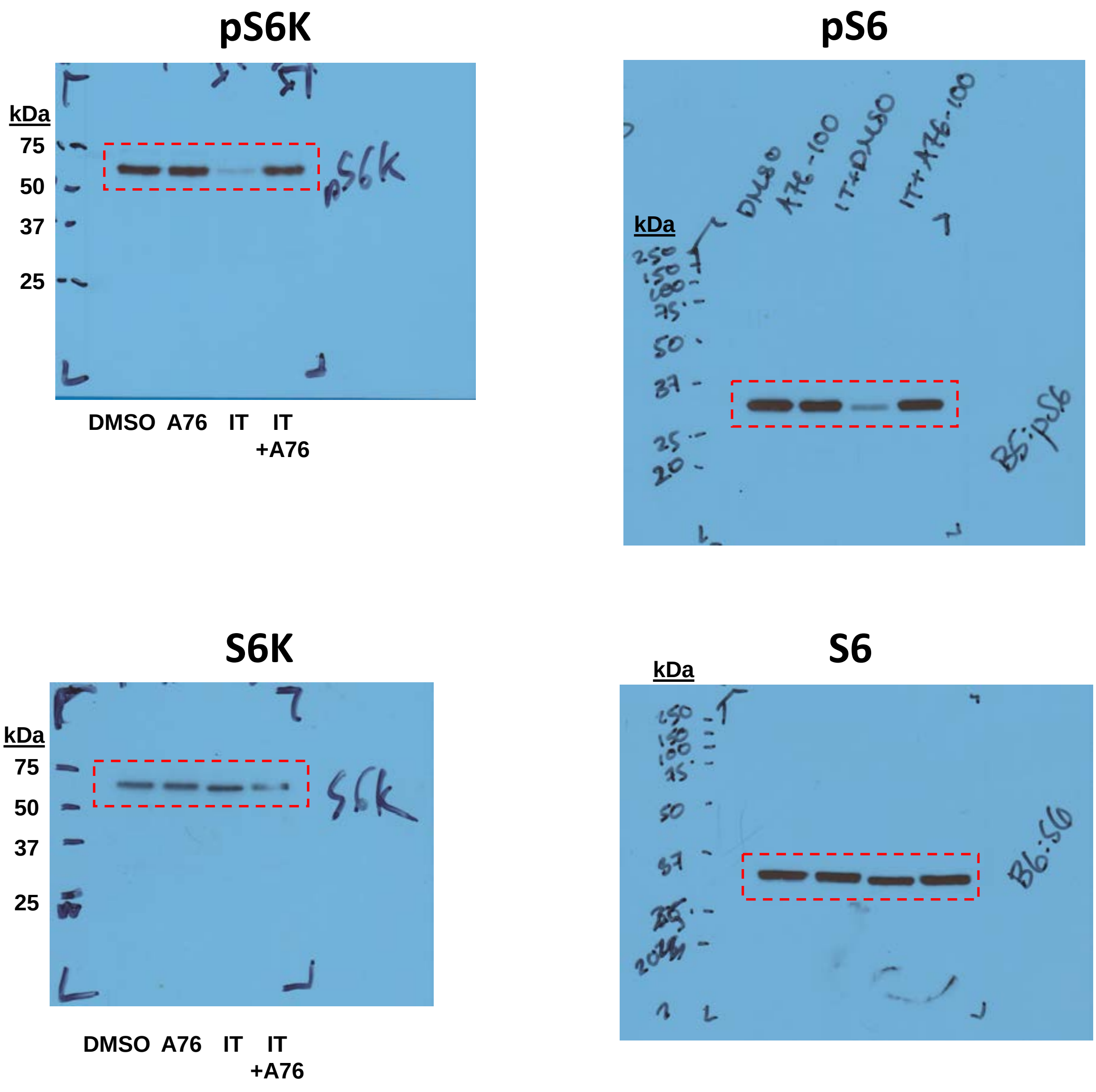

Abbreviations: A76, A-769662. IT, IFNγ/TNFα

Figure 6 - Panel E

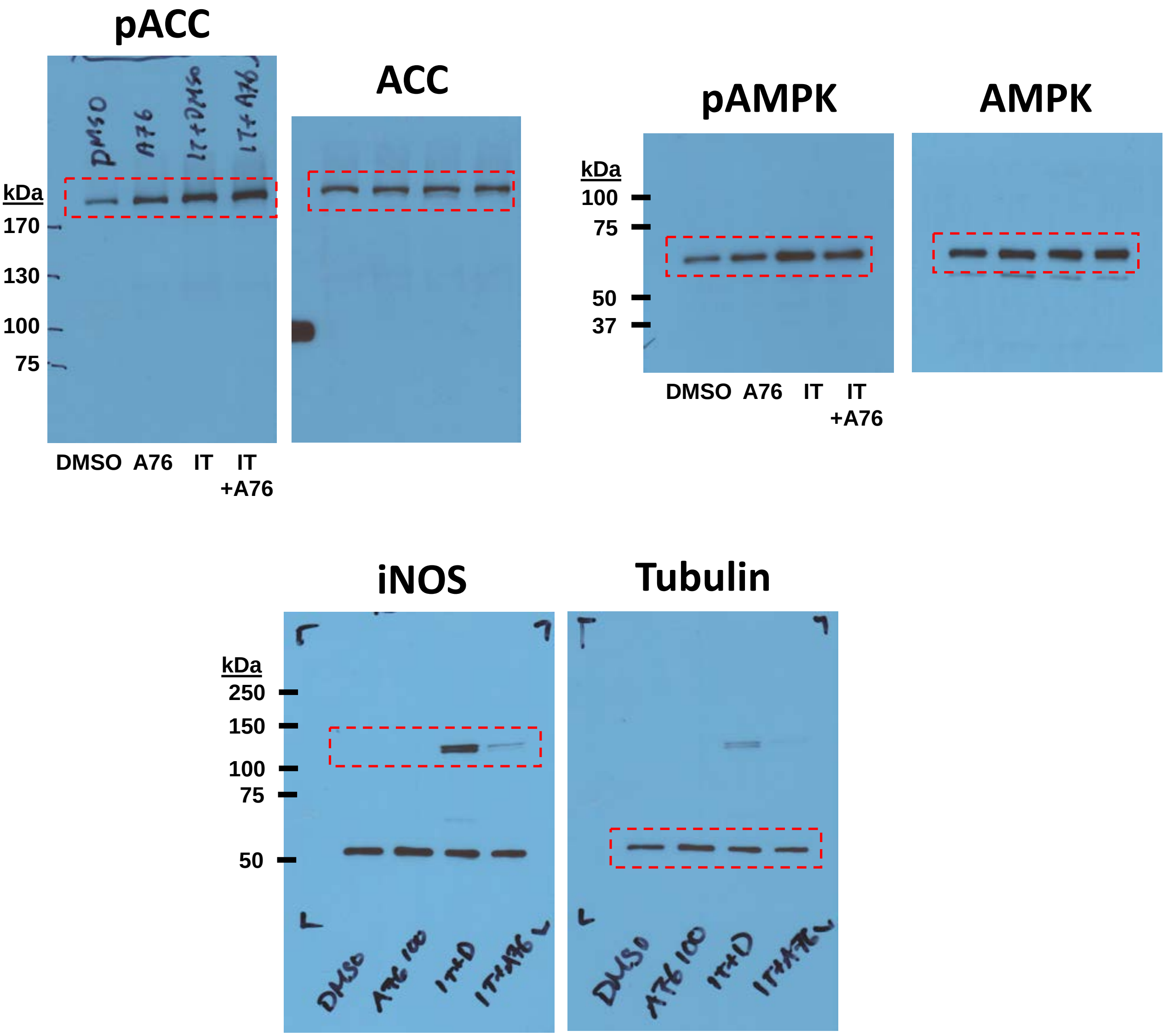

Abbreviations: A76, A-769662. IT, IFNγ/TNFα
