# Supplementary figures and images for: The AMPK agonist 5‐aminoimidazole‐4‐carboxamide ribonucleotide (AICAR), but not metformin, prevents inflammation‐associated cachectic muscle wasting
Source: EMBO Mol Med. 2018 May 29;10(7):e8307. doi: 10.15252/emmm.201708307 (PMC6034131; doi:10.15252/emmm.201708307)

Figure 7 - Panel B

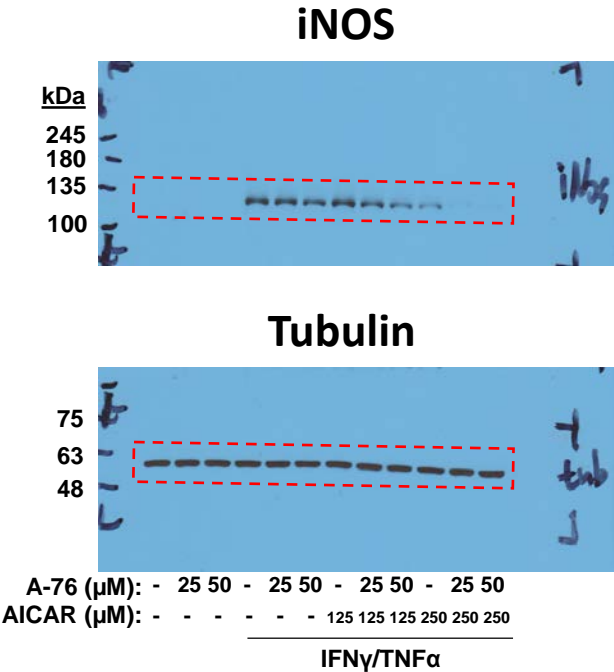

Abbreviations: A76, A-769662.

Figure 7 - Panel C

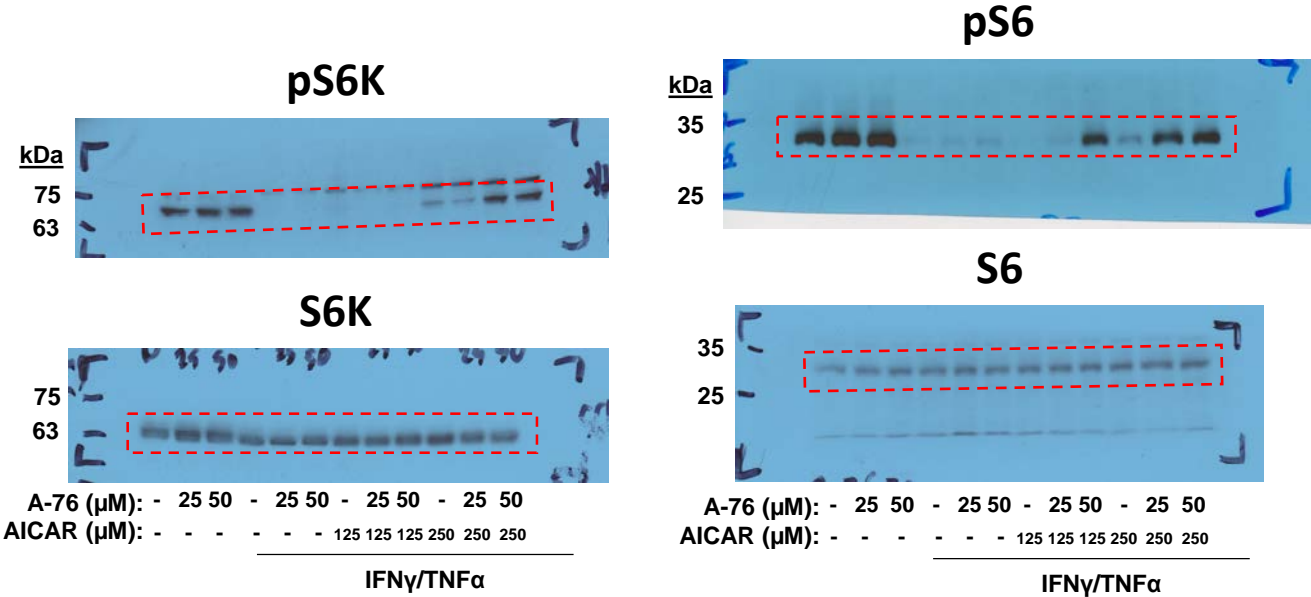

Abbreviations: A76, A-769662.

Supplement: Supplementary file 9 — Source Data for Figure 7 [file EMMM-10-e8307-s008.pdf]

**Figure 9 - Panel A**

**PF**

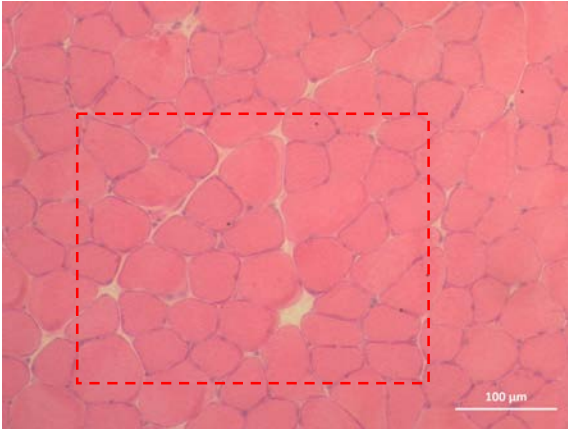

**LPS**

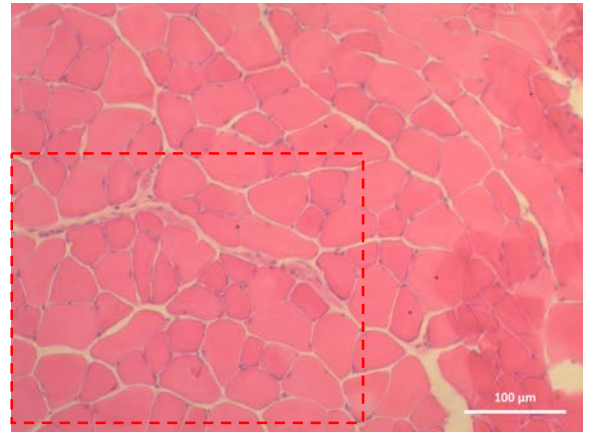

**LPS + A**

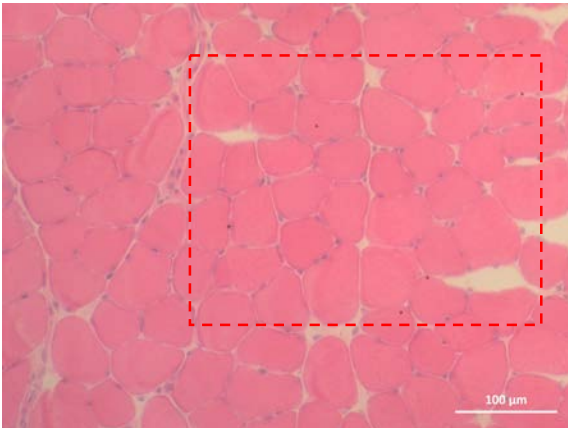

**LPS + M**

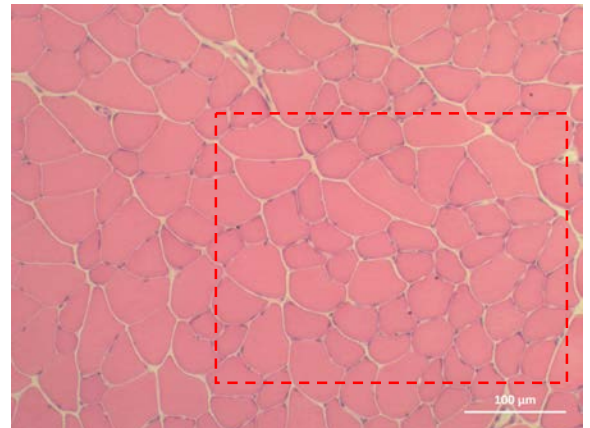

Abbreviations: PF, pair-fed. A, AICAR. M, metformin.

Supplement: Supplementary file 11 — Source Data for Figure 9 [file EMMM-10-e8307-s010.pdf]
