# Supplementary material for: The AMPK agonist 5‐aminoimidazole‐4‐carboxamide ribonucleotide (AICAR), but not metformin, prevents inflammation‐associated cachectic muscle wasting
Source: EMBO Mol Med. 2018 May 29;10(7):e8307. doi: 10.15252/emmm.201708307 (PMC6034131; doi:10.15252/emmm.201708307)
Supplement: Supplementary file 10 — Source Data for Figure 8 [file EMMM-10-e8307-s009.pdf]

Figure 8 - Panel A

Saline

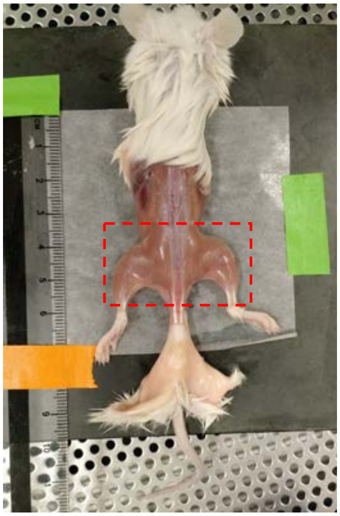

C26

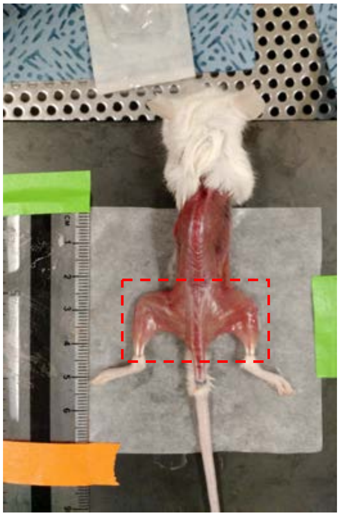

C26 + A

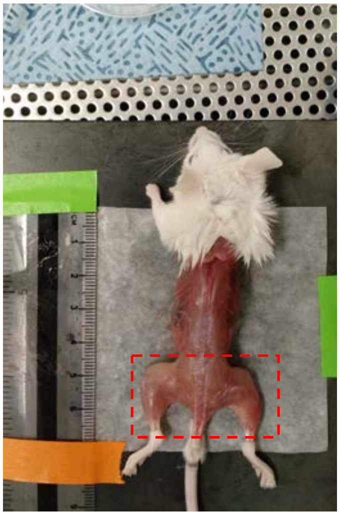

C26 + M

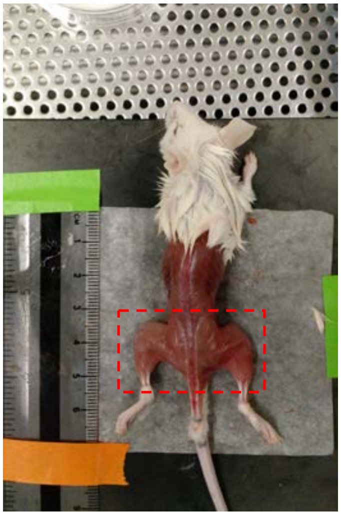

Abbreviations: A, AICAR. M, metformin.

Figure 8 - Panel B

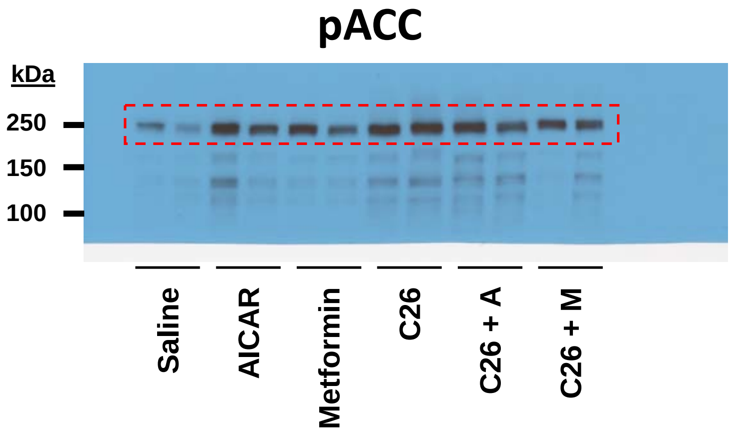

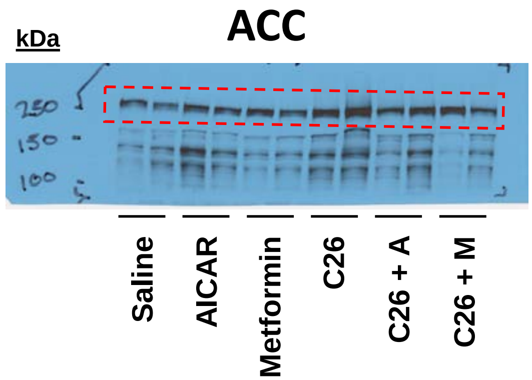

Abbreviations: A, AICAR. M, metformin.

Figure 8 - Panel C

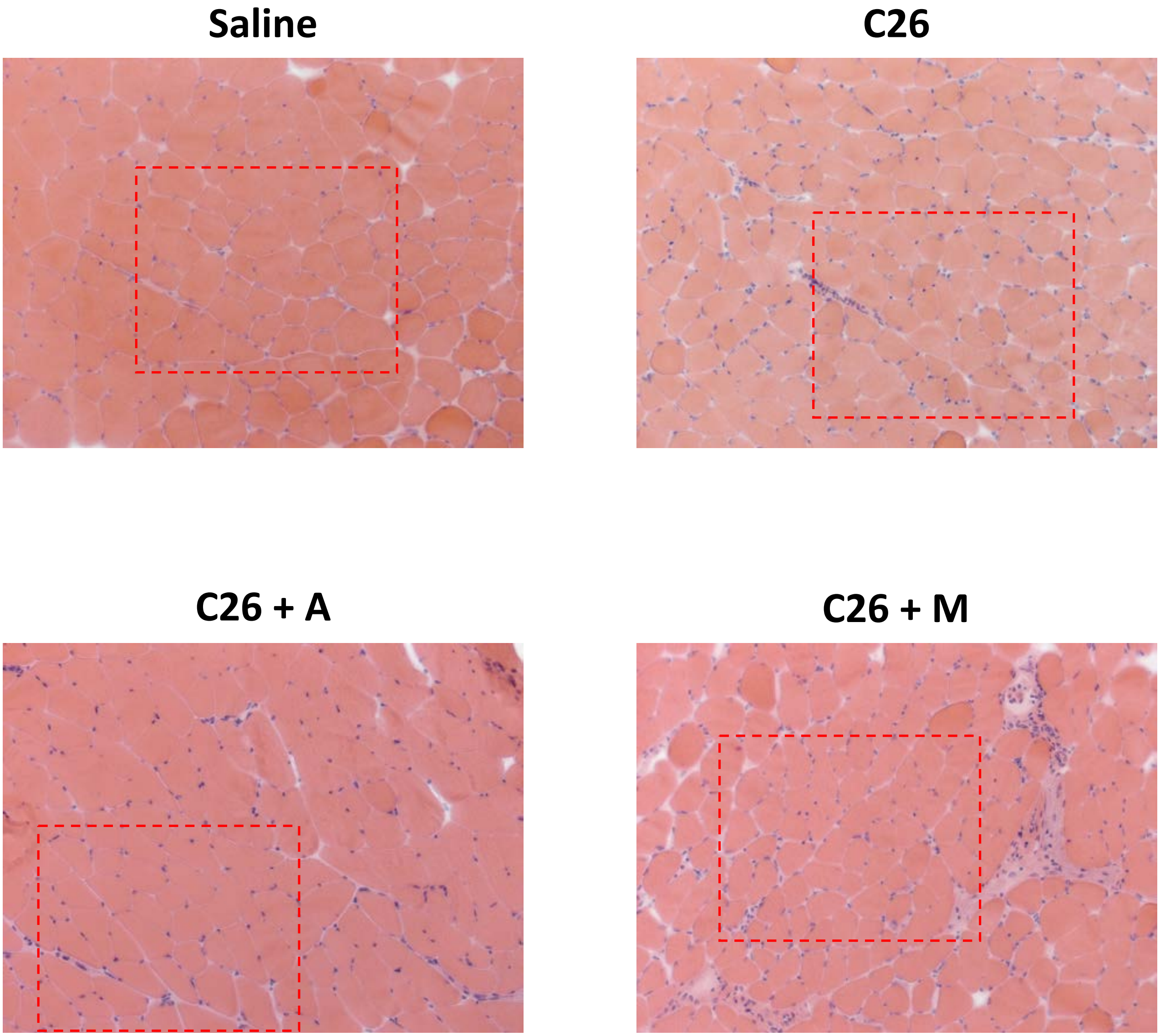

Abbreviations: A, AICAR. M, metformin.
